# Blood and urine early treatment response biomarkers in HIV-associated disseminated tuberculosis

**DOI:** 10.4102/sajhivmed.v26i1.1664

**Published:** 2025-04-09

**Authors:** Linda Boloko, Marcia Vermeulen, Bianca Sossen, Abulele Bekiswa, Phiona E. Namale, Chad Centner, Robert J. Wilkinson, Charlotte Schutz, Graeme Meintjes, David A. Barr

**Affiliations:** 1 Centre for Infectious Diseases Research in Africa (CIDRI-Africa), Institute of Infectious Disease and Molecular Medicine, University of Cape Town, Cape Town, South Africa; 2 Department of Medicine, University of Cape Town, Cape Town, South Africa; 3 Division of Infectious Diseases and HIV Medicine, Faculty of Health Sciences, University of Cape Town, Cape Town, South Africa; 4 University of Cape Town and National Health Laboratory Service, Cape Town, South Africa; 5 The Francis Crick Institute, London, United Kingdom; 6 Department of Infectious Disease, Faculty of Medicine, Imperial College London, London, United Kingdom; 7 Blizard Institute, Faculty of Medicine and Dentistry, Queen Mary University of London, London, United Kingdom; 8 Department of Infectious Diseases, Queen Elizabeth University Hospital, Glasgow, United Kingdom

**Keywords:** HIV-associated tuberculosis, biomarkers, bacteraemia, lipoarabinomannan, urine

## Abstract

**Background:**

Treatment response biomarkers are needed in the care of patients hospitalised with HIV-associated tuberculosis (TB).

**Objectives:**

We describe the changes in bacillary load during early treatment using quantitative and semi-quantitative measures of *Mycobacterium tuberculosis* in blood and urine.

**Method:**

We collected serial blood and urine samples at multiple timepoints in consenting adult patients with HIV and positive urine lipoarabinomannan (LAM), admitted to Mitchells Plain Hospital, Cape Town. Blood and urine Xpert Ultra, mycobacterial blood culture and urine LAM were performed. Survival analysis and mixed-effects modelling were used to determine time to a negative test, and to give the predicted probability of a positive test at the different timepoints.

**Results:**

Sixteen participants, predominantly male (63%), with median age 39 years (interquartile range [IQR] 36–43), and CD4 count 27 cells/mm^3^ (IQR 8–83) were included. At day 14, urine LAM, urine Xpert Ultra and blood Xpert Ultra remained positive in between 75% and 86% of the participants. A mixed-effects model predicted a decline in ordinal values of urine Xpert Ultra (cycle threshold), blood Xpert Ultra (cycle threshold) and blood culture (time-to-positivity) in response to anti-TB treatment. Conversely, urine LAM grade intensity increased over the 14 days.

**Conclusion:**

*M. tuberculosis* DNA was detectable in urine and blood in decreasing quantity up to 14 days of standard treatment in patients with HIV-associated TB. Urine Alere LAM showed an increasing grade intensity during this period. Further research in larger groups and extended periods are needed to assess relation to clinical outcomes.

**What this study adds:** In this study in patients with HIV-associated disseminated and extra-pulmonary tuberculosis (TB), we show the potential value of Xpert MTB/RIF Ultra as a non-sputum-based treatment response biomarker.

## Introduction

*Mycobacterium tuberculosis* is the leading cause of sepsis and death in people living with HIV (PLWH) in sub-Saharan Africa, and tuberculosis (TB) is disseminated at death in 88% of HIV-associated TB cases.^1,2,3,4^ The non-specific clinical presentation, often mimicking other clinical syndromes, may lead to delay in diagnosis and treatment, increasing mortality.^5^ Current therapeutic and treatment response strategies follow those of outpatient pulmonary TB in HIV-negative patients.^6,7^ Sputum is often difficult to obtain in critically ill patients admitted with HIV-associated TB.^8,9,10^ Similarly, studies investigating novel drug regimens still rely heavily on sputum-based culture conversion strategies, thus excluding patients unable to produce sputum. Furthermore, treatment response assessment in programmatic settings is performed at week 8, a timepoint where over 20% of patients with HIV-associated TB may have died.^2,11,12,13^

Several factors are associated with mortality in HIV-associated TB including disseminated disease, drug resistance, sepsis syndrome and an immune profile characterised by innate immune activation.^2,14,15^ Patients with disseminated TB are at highest risk of mortality during the first 2 weeks of treatment.^2^ Current treatment response monitoring strategies in disseminated and extra-pulmonary TB are based on clinical features such as weight gain as a surrogate marker for treatment efficacy, and, similar to smear microscopy, are often evaluated at the end of the intensive phase.^16,17^ C-reactive protein is an alternative treatment response biomarker but limited by poor specificity where it may paradoxically increase especially in PLWH who have other opportunistic infections and who may develop immune reconstitution inflammatory syndrome after initiating antiretroviral therapy.^18^

Urine and blood-based treatment response biomarkers could provide a measure of treatment efficacy in treatment intensification and drug development clinical trials, as well as in treatment response in clinical care. Improved understanding of the decline of *M. tuberculosis* in blood or urine during standard treatment could be a novel TB pharmacodynamic parameter and could inform the development of novel pathogen and host-directed therapies in this patient population. In this prospective cohort study, we serially quantified *M. tuberculosis* in blood and urine over a 14-day period in patients admitted to hospital with HIV-associated disseminated TB, using the urine Alere lipoarabinomannan (LAM) assay, and Xpert MTB/RIF (rifampicin) Ultra performed on urine and blood and mycobacterial blood cultures. We (1) described agreement between test replicates and between different tests, (2) determined how long TB bacilli remained detectable by different tests during the first 14 days of treatment, and (3) observed the change in the bacillary load in blood and urine using the semi-quantitative urine Alere LAM grading, quantitative Xpert MTB/RIF Ultra cycle thresholds (urine and blood), and mycobacterial blood culture time-to-positivity (TTP).

## Research methods and design

### Study design and population

We prospectively recruited PLWH (18 years or older) with suspected TB admitted to Mitchells Plain Hospital (Cape Town, South Africa) between November 2020 and August 2021. We included patients who were positive by urine Alere LAM (Determine™ TB LAM Ag, Abbott Laboratories, Lake Bluff, Illinois, United States) or FUJIFILM SILVAMP TB LAM (FujiLAM; Fujifilm, Tokyo, Japan). We further enriched recruitment using high predicted probability of *M. tuberculosis* blood stream infection: a bespoke prediction model (https://davidadambarr.shinyapps.io/lindaboloko/) developed using prior data^2,3^ of readily available baseline clinical variables accessed via a smartphone application at the patient’s bedside; patients with ≥ 50% predicted probability of positive TB blood culture were asked to participate. We excluded patients who received any anti-TB treatment prior to enrolment, had haemoglobin < 5 g/dL or symptomatic anaemia with haemoglobin 5 g/dL – 8 g/dL. Participants were followed up for blood and urine collection at day 0 before standard anti-TB treatment (rifampicin, isoniazid, pyrazinamide, ethambutol), at day 1, day 3, day 7, day 14 on anti-TB treatment, and subsequently at day 28 for vital status. This was a pilot study and the sample size of 16 patients was not powered for hypothesis testing.

### Study procedures

At baseline (day 0), we collected fresh spot urine in 50 mL falcon tubes with varying volumes between participants, prior to initiation of anti-TB treatment. Urine Alere LAM testing was done at the bedside and read at 25 min using reference cards as per manufacturer’s instruction. Urine was then aliquoted into separate volumes for storage at –80 °C and for urine Xpert MTB/RIF Ultra (Cepheid, Sunnyvale, California, United States) testing done by the National Health Laboratory Services. Similarly, blood was collected at baseline prior to institution of anti-TB treatment in 4 mL K_2_EDTA (dipotassium ethylenediaminetetraacetic acid; Becton, Dickinson and Company, Franklin Lakes, New Jersey, United States) and 6 mL heparin sodium (Becton, Dickinson and Company, Franklin Lakes, New Jersey, United States) tubes. Ethylenediaminetetraacetic acid (EDTA) blood was separated into plasma for storage at –80°C and the red cell pellet used for performing blood Xpert Ultra. We separated heparin sodium blood for plasma storage and the red blood cell pellet for mycobacterial blood culture. Red blood cell lysis buffer was added to the pellet, incubated at room temperature, centrifuged, and inoculated into a blood culture bottle, reducing potential antimicrobial drug carry-over from plasma. TB blood cultures were performed by liquid culture (BACTEC Myco F/Lytic; Becton, Dickinson and Company, Franklin Lakes, New Jersey, United States) and *M. tuberculosis* was identified by GenoType MTBDR*plus* line-probe assay (Hain Lifescience GmbH, Nehren, Germany). Serial follow-up samples were collected and processed as described above on days 1, 3, 7 and 14. Serial urine LAM measurement was done on frozen samples after completion of the clinical study.

### Statistical analysis

The minimum cycle threshold value of the four Xpert Ultra *rpoB* probes was used, with ‘trace’ positive samples imputed by modelling the relationship between *rpoB* and IS6110-1081. We assessed reproducibility of assay results by examining concordance of each possible pairwise comparison of technical replicates (the same assay performed on replicate samples from the same participant at the same timepoint), summarising agreement using Cohen’s Kappa statistic. We also assessed correlation of semi-quantitative results (cycle threshold [Ct] values of Xpert Ultra; and blood culture TTP) for technical replicate pairs. We similarly assessed agreement and correlation between different assays using all available test pairs from same participant-timepoints.

Association between Ct values, TTP, or LAM grade and mortality was assessed using Wilcoxon rank sum testing (ignoring nesting of replicates). Confidence intervals for proportions were derived from binomial proportion tests. To assess change in bacillary load in blood and urine following treatment, results for each assay were converted to an ordinal scale ranging from 0 to 4. A negative result was assigned 0, and values 1–4 were assigned based on quartiles, such that a value of 4 indicated highest bacillary load detection (lowest Ct, lowest TTP, or highest LAM grade). We then estimated the effect of days on treatment on ordinal category using linear mixed-effects models, including random effects on intercept and slope by participant to account for nesting of replicates. These models were fit with the Bayesian Regression Models using ‘Stan’ (BRMS) package in R using non-informative priors. Predicted ordinal category from these model fits was then used as a time-varying predictor variable for mortality in a Cox proportional hazards model, to assess for evidence that bacillary load exposure over time of follow-up was associated with mortality hazard. All analysis was done using RStudio v1·2·5033 (RStudio, PBC, Boston, Massachusetts, United States).

### Role of the funding sources

The funders of the study had no role in study design, data collection, data analysis, data interpretation, or writing of the report.

### Ethical considerations

Ethical clearance to conduct this study was obtained from the University of Cape Town Human Research Ethics Committee (reference no.: HREC 645/2021). This study was approved by the Institutional Review Board. All participants provided written informed consent. All data were treated with confidentiality.

## Results

### Baseline characteristics

**FIGURE 1:**
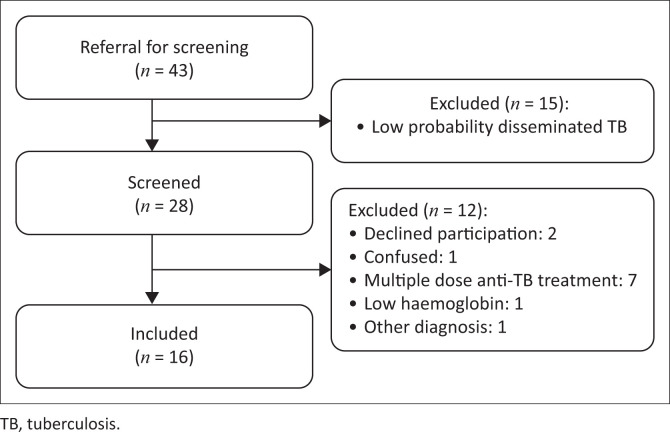
Inclusion and exclusion from study.

Sixteen participants who were either Alere LAM or FujiLAM positive were enrolled in the study (Figure 1). Three participants were lost to follow-up at day 1 (*n* = 1) and day 7 (*n* = 2). The median age was 39 years (interquartile range [IQR] 36–43), median CD4 count was 27 cells/mm^3^ (IQR 8–83), and predominantly male participants (63%) were included in the study. Six (38%) participants reported taking antiretroviral therapy at enrolment. Fifteen (94%) participants had microbiological evidence of TB. There were five in-hospital deaths (31%), with median time to death being 7 days (IQR 2–9). The baseline characteristics are presented in Table 1 and Online Appendix Table 1-A1.

**TABLE 1:** Baseline characteristics (*N* = 16).

Variable	Median	IQR	*n*	%
Age (years)	39	36–43	-	-
**Sex**				
Female	-	-	6	38
Male	-	-	10	63
Current ART use	-	-	6	38
CD4 count (cells/mm^3^)	27	8–83	-	-
Previous tuberculosis	-	-	9	56
White cell count (×10^9^/L)	6.1	3.9–9.2	-	-
Haemoglobin (g/dL)	9.1	7.6–10.5	-	-
Platelets (×10^9^/L)	203	121–378	-	-
Creatinine (µmol/L)	111	73–374	-	-
*Mycobacterium tuberculosis* blood stream infection prediction score†	0.49	0.42–0.62	-	-
**Symptoms**				
Cough	-	-	6	38
Fever	-	-	8	50
Loss of appetite	-	-	13	81
Malaise	-	-	16	100
Night sweats	-	-	4	25
Weight loss	-	-	14	88
**Baseline Alere LAM grade**				
Negative	-	-	3	19
1+	-	-	0	0
2+	-	-	2	12
3+	-	-	5	31
4+	-	-	6	38

IQR, interquartile range; ART, antiretroviral therapy; LAM, lipoarabinomannan.

† , Variables included in the model: sodium, creatinine, lactate, haemoglobin, lymphocyte, CD4 count, Alere LAM, heart rate, ECOG (Eastern Cooperative Oncology Group), peripheral/mediastinal lymph nodes, miliary chest X-ray.

### Detection of disseminated tuberculosis

Three technical replicates at each of five timepoints for the 16 participants yielded valid results: for blood Xpert Ultra samples, 78% (187/240), urine Xpert Ultra 71% (171/240), and mycobacterial blood culture 69% (165/240). Urine LAM had a valid result in 55% (44/80) (Online Appendix Figure 1-A1). Missing results were because of early death, loss to follow-up, urine unavailability, and technical factors including contamination. There was strong agreement within technical replicates of each assay, and Cohen’s Kappa similar for the three methods (0.67 for blood Xpert Ultra, 0.71 for urine Xpert Ultra, and 0.68 for mycobacterial blood culture) (Online Appendix Table 2-A1). In addition, there was moderate agreement between blood Xpert Ultra and urine Xpert Ultra (Cohen’s Kappa 0.32, 95% confidence interval [CI] 0.24–0.40), and slight agreement for blood culture and urine Xpert Ultra (Cohen’s Kappa 0.09, 95% CI 0.05–0.13) (Online Appendix Table 2).

### Quantitative and semi-quantitative measures of bacillary load

**FIGURE 2:**
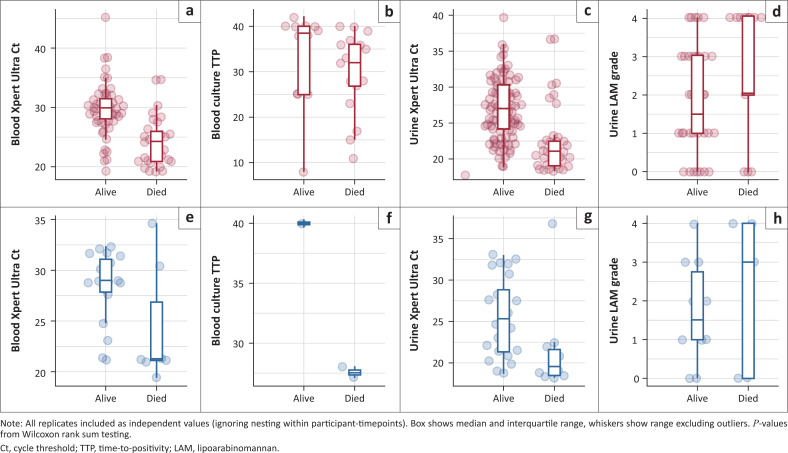
Association between quantitative and semi-quantitative bacilli load readouts and mortality. (a-d) All available samples (all replicates, all timepoints): (a) Blood Xpert Ultra cycle threshold (*P* < 0.001), (b) Blood culture time-to-positivity (*P* = 0.14), (c) Urine Xpert Ultra cycle threshold (*P* ≤ 0.001), (d) Urine lipoarabinomannan grade (*P* = 0.038). (e-h) Pre-treatment samples (all replicates, day 0): (e) Blood Xpert Ultra cycle threshold (*P* = 0.041), (f) Blood culture time-to-positivity (*P* = 0.67), (g) Urine Xpert Ultra cycle threshold (*P* = 0.0062), (h) Urine lipoarabinomannan grade (*P* = 0.66).

The median cycle threshold prior to treatment was 29 (25–30) for blood Xpert Ultra and 23 (19–30) for urine Xpert Ultra; median time to blood culture positivity was 28 days (IQR 24–30) and the median LAM grade intensity was 3+ (IQR 2–4). Correlation of semi-quantitative results (Ct or TTP) within technical replicates of the same assay ranged from *r* = 0.92 for paired, pre-treatment blood Xpert Ultra sample Ct values, to *r* = 0.77 for blood culture TTP from paired samples taken after day 0 of treatment (Online Appendix Figure 2-A1). Correlations between the three assays (comparing blood vs urine Xpert Ultra Ct; blood culture TTP vs blood Xpert Ultra Ct; and urine Xpert Ultra Ct vs blood culture TTP) were similarly high at day 0. However, correlations were substantially lower for samples taken after participants were on TB treatment (Online Appendix Figure 2). For both blood and urine Xpert Ultra, the distribution of Ct values (across all replicates and timepoints) was strongly associated with study outcome: lower Ct values, indicating higher bacillary load, being found in the participants who died. Such an association was less clear for blood culture TTP and urine LAM grade (Figure 2).

### Changes in blood and urine biomarkers in response to treatment

**FIGURE 3:**
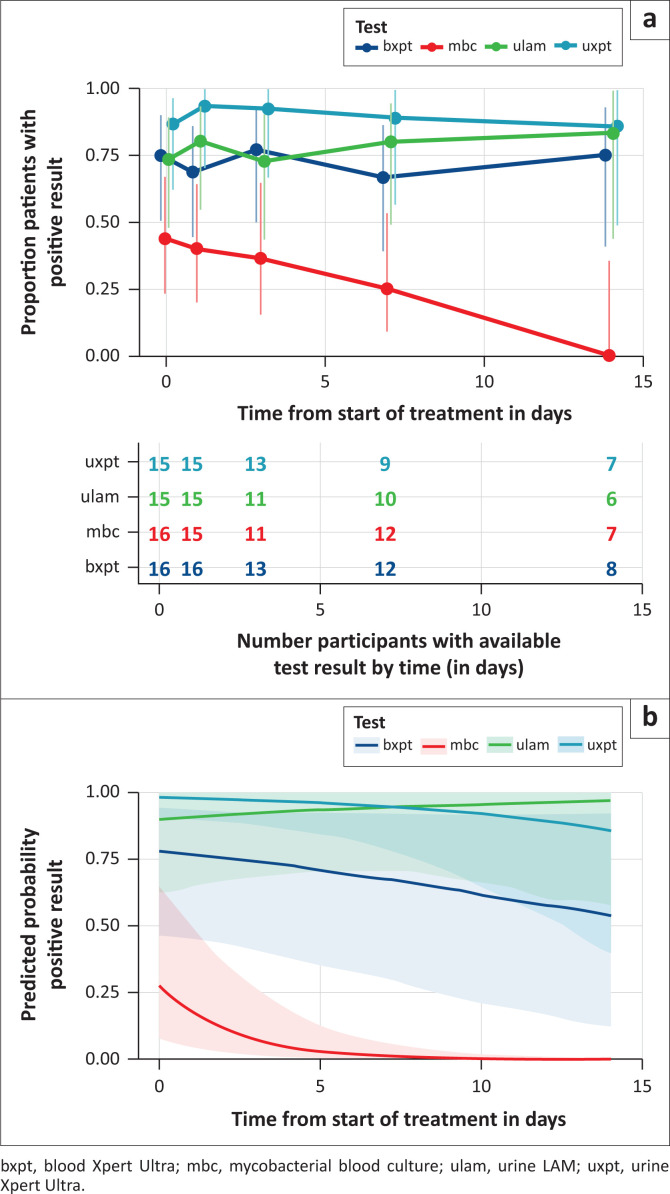
Proportion positive and remaining positive during the study follow-up period. (a) Proportion of participants with at least one positive result among replicates at timepoint, with 95% confidence interval (CI) shown with error bars and numbers ‘at risk’ (participants with at least one valid test result at this timepoint) in Table. (b) Predicted probabilities of a positive test result from logistic regression mixed-effects model with replicates nested within participant-timepoints and a random intercept and slope included in model. Shaded areas give 95% CI for mean predicted probability.

At baseline, at least one positive urine LAM, urine Xpert Ultra, blood Xpert Ultra, or mycobacterial blood culture was found in 11/15 (73%), 13/15 (87%), 12/16 (75%) and 7/16 (44%) participants, respectively. Blood and urine Xpert Ultra, and urine LAM remained detectable in a similar proportion across all time points, with 6/8 participants remaining blood Xpert Ultra positive at day 14 (75% CI 41–93). In contrast, the proportion with a positive blood culture fell progressively over this period, with no TB blood stream infection detected by culture on day 14 (Figure 3).

We assessed the change in bacillary load in blood and urine following treatment with each assay converted to an ordinal scale ranging from 0 (negative test) to 4 (lowest Ct or TTP, or highest LAM grade). A linear mixed-effects model was used to assess for an effect of time on treatment on assay ordinal scale value. There was interindividual variability on all the assays; however, on average, ordinal category decreased with days on treatment for blood Xpert Ultra, urine Xpert Ultra and mycobacterial blood culture during the first 14 days of anti-TB treatment.

**FIGURE 4:**
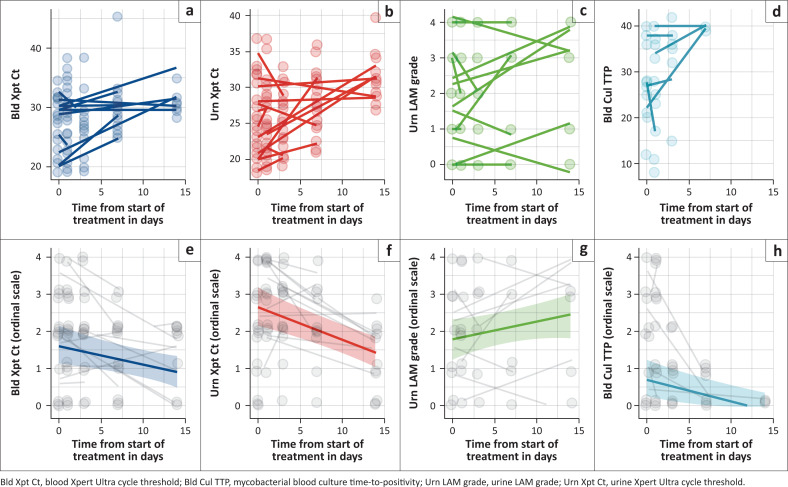
Measures of bacilli load over time on treatment. (a–d) Semi-quantitative results (blood and urine Xpert Ultra Ct, mycobacterial blood culture TTP, and urine LAM grade) over time, with a line of best fit per participant. In (e–h), these results have been adapted to an ordinal scale, with 1 representing lowest bacilli load (highest Ct or TTP, lowest LAM grade), and 4 representing highest bacilli load (lowest Ct or TTP, highest LAM grade), and with negative test results included as 0 on the scale. Predicted ordinal level from a linear mixed-effects model (mean value) is shown with coloured line and shaded 95% CI.

By contrast, urine LAM grade intensity increased slightly (Figure 4). A Cox proportional hazards model with ordinal category as a time-varying predictor of mortality showed positive hazard ratios for blood Xpert Ultra, urine Xpert Ultra and mycobacterial blood culture, and a negative hazard ratio for urine LAM, but none of these effect sizes was statistically significant (Online Appendix Table 3-A1).

## Discussion

In our study, *M. tuberculosis* DNA was detectable by Xpert Ultra in blood and urine in over two-thirds of patients with disseminated HIV-associated TB up to 14 days after starting standard anti-TB treatment. *M. tuberculosis* was detected by liquid culture in blood up to day 7 of anti-TB treatment. We also showed that there is a decline bacillary load as measured by Xpert Ultra cycle threshold and blood culture TTP, while, conversely, urine LAM concentrations appear to increase during the first 14 days of treatment.

In routine management of pulmonary TB, treatment response is assessed by conversion of sputum TB tests from positive to negative. We demonstrate that TB can be detected and quantified in urine and blood up to 14 days into treatment, suggesting that an analogous approach may be possible for disseminated TB treatment using these novel diagnostic platforms. We suggest that Xpert Ultra could have utility in the monitoring of treatment response in the first 2 weeks, when mortality is highest in HIV-associated TB.

Tools to detect extra-pulmonary *M. tuberculosis* are advancing our understanding of the TB disease spectrum.^19,20^ We found good reliability of Xpert Ultra agreement and correlation across technical replicates to detect and quantify TB in blood and urine, supporting the use of these tools when studying the pathophysiology of advanced HIV-associated TB. There was interpatient variability in the dynamics of bacillary load measures across the different assays. Heterogeneity in sputum conversion in response to treatment is known to occur and is associated with HIV status, baseline smear status, and presence or absence of cavities. Similarly, variation in blood and urine may occur because of pharmacokinetics, immune response, and bacillary load.^21^ Further research is needed to interrogate these associations. Pre-treatment, higher bacillary load measured by blood and urine Xpert Ultra was associated with mortality persisting through all timepoints. Agreement and correlation of bacilli readouts between blood and urine in this study suggests that these compartments have some continuity in disseminated infection, either because renal TB disease is a frequent complication of bateraemia and/or because both blood and urine bacillary load are markers of underlying total systemic bacillary load.^22,23^

In contrast to the other assays, our data did not show a decrease in the urine LAM band intensity in the follow-up period, in keeping with previous results using optical density measure showing stability in the first 2 weeks with subsequent decrease over 24 weeks.^24,25,26^ This limits the use of this platform for early treatment monitoring. We hypothesise that during early treatment there is ongoing release of LAM antigen and, similarly, *M. tuberculosis* DNA from dying mycobacteria, particularly in those patients with high disease load such as disseminated TB and possible renal involvement. It is plausible that, as with cell-free DNA, *M. tuberculosis* DNA is released via apoptosis and necrosis.^27^ This is likely exaggerated during anti-TB drug treatment. Heavy bacillary load in tissues may result in bacterial antigen and DNA seeding into the bloodstream from tissues where it can be recovered by Xpert Ultra. Similarly, release into the renal tract is recoverable by urine Xpert Ultra.^24^

Ct values were lower in urine, indicating greater DNA concentrations compared to blood. Several mechanisms may be responsible for this difference. Firstly, there may be direct release into the urine from renal tissue in addition to DNA filtered through the glomerulus, increasing recovery by urine Xpert Ultra. Secondly, cell-free DNA in blood has a half-life of approximately 2 h after clearance by DNAses, macrophage degradation, and renal excretion.^28,29^ Finally, technical factors such as our concentration methods may be better in urine than blood, where additional wash steps may result in loss of DNA material.

### Implications

Our findings have several potential implications for the management of HIV-associated TB admitted to hospital. Firstly, given the detection of *M. tuberculosis* DNA in blood and urine at 14 days despite the decline in bacillary load, it is possible that the current standard first-line treatment initially developed for pulmonary TB may not be adequate for initial clearance of infection in patients with a high bacillary load in the context of disseminated disease. Secondly, Xpert Ultra quantitative outputs may potentially be used to establish treatment intensification targets as surrogate markers of treatment efficacy.

### Limitations

Our study had several limitations. Firstly, this was a pilot study with a small sample size and requires a larger study to confirm these findings and their associations with clinical outcomes. Secondly, we detected *M. tuberculosis* using Xpert MTB/RIF Ultra, which does not differentiate between live and dead bacteria. However, detection by blood culture strengthens the likelihood that Xpert Ultra detected viable *M. tuberculosis.* Thirdly, our blood culture method to remove antibiotics in longitudinal blood cultures suffered a technical-related contamination (the blood cultures were contaminated because of a contaminated batch of pre-processing reagents), disallowing detection of *M. tuberculosis* beyond 7 days. Furthermore, the volume of blood collected for mycobacterial culture was not standardised and could have affected the results. However, this finding is still novel, as no previous studies have detected *M. tuberculosis* in blood culture beyond 72 h of treatment.

## Conclusion

Blood and urine Xpert Ultra quantitative outputs may have utility as non-sputum-based treatment response biomarkers in patients with HIV-associated TB, and need further research in larger cohorts and extended periods. Furthermore, longitudinal measure of these biomarkers may provide more insight into the pathogenesis and pathophysiology of disseminated tuberculosis.

## References

[1] Jacob ST, Moore CC, Banura P, et al. Severe sepsis in two Ugandan hospitals: A prospective observational study of management and outcomes in a predominantly HIV-1 infected population. PLoS One. 2009;4(11):e7782. 10.1371/journal.pone.0007782 19907656 PMC2771355

[2] Schutz C, Barr D, Andrade BB, et al. Clinical, microbiologic, and immunologic determinants of mortality in hospitalized patients with HIV-associated tuberculosis: A prospective cohort study. PLoS Med. 2019;16(7):e1002840. 10.1371/journal.pmed.1002840 31276515 PMC6611568

[3] Gupta RK, Lucas SB, Fielding KL, Lawn SD. Prevalence of tuberculosis in post-mortem studies of HIV-infected adults and children in resource-limited settings: A systematic review and meta-analysis. AIDS. 2015;29(15):1987–2002. 10.1097/QAD.0000000000000802 26266773 PMC4568896

[4] Lewis JM, Feasey NA, Rylance J. Aetiology and outcomes of sepsis in adults in sub-Saharan Africa: A systematic review and meta-analysis. Crit Care. 2019;23(1):212. 10.1186/s13054-019-2501-y 31186062 PMC6558702

[5] Barr DA, Lewis JM, Feasey N, et al. Mycobacterium tuberculosis bloodstream infection prevalence, diagnosis, and mortality risk in seriously ill adults with HIV: A systematic review and meta-analysis of individual patient data. Lancet Infect Dis. 2020;20(6):742–752. 10.1016/S1473-3099(19)30695-4 32178764 PMC7254058

[6] Rockwood N, Du Bruyn E, Morris T, Wilkinson RJ. Assessment of treatment response in tuberculosis. Expert Rev Respir Med. 2016;10(6):643–654. 10.1586/17476348.2016.116696 27030924 PMC4949330

[7] Moule MG, Cirillo JD. Mycobacterium tuberculosis dissemination plays a critical role in pathogenesis. Front Cell Infect Microbiol. 2020;10:65. 10.3389/fcimb.2020.00065 32161724 PMC7053427

[8] Kerkhoff AD, Barr DA, Schutz C, et al. Disseminated tuberculosis among hospitalised HIV patients in South Africa: A common condition that can be rapidly diagnosed using urine-based assays. Sci Rep. 2017;7(1):10931. 10.1038/s41598-017-09895-7 28883510 PMC5589905

[9] Lawn SD, Kerkhoff AD, Burton R, et al. Diagnostic accuracy, incremental yield and prognostic value of determine TB-LAM for routine diagnostic testing for tuberculosis in HIV-infected patients requiring acute hospital admission in South Africa: A prospective cohort. BMC Med. 2017;15(1):67. 10.1186/s12916-017-0822-8 28320384 PMC5359871

[10] Sossen B, Meintjes G. Development of accurate non-sputum-based diagnostic tests for tuberculosis: An ongoing challenge. Lancet Glob Health. 2023;11(1):e16–e17. 10.1016/S2214-109X(22)00513-7 36521945

[11] Gupta-Wright A, Corbett EL, Van Oosterhout JJ, et al. Rapid urine-based screening for tuberculosis in HIV-positive patients admitted to hospital in Africa (STAMP): A pragmatic, multicentre, parallel-group, double-blind, randomised controlled trial. Lancet. 2018;392(10144):292–301. 10.1016/S0140-6736(18)31267-4 30032978 PMC6078909

[12] Peter JG, Zijenah LS, Chanda D, et al. Effect on mortality of point-of-care, urine-based lipoarabinomannan testing to guide tuberculosis treatment initiation in HIV-positive hospital inpatients: A pragmatic, parallel-group, multicountry, open-label, randomised controlled trial. Lancet. 2016;387(10024):1187–1197. 10.1016/S0140-6736(15)01092-2 26970721

[13] Gupta-Wright A, Fielding K, Wilson D, et al. Tuberculosis in hospitalized patients with human immunodeficiency virus: Clinical characteristics, mortality, and implications from the rapid urine-based dcreening for tuberculosis to reduce AIDS related mortality in hospitalized patients in Africa. Clin Infect Dis. 2020;71(10):2618–2626. 10.1093/cid/ciz1133 31781758 PMC7744971

[14] Boloko L, Schutz C, Sibiya N, et al. Xpert ultra testing of blood in severe HIV-associated tuberculosis to detect and measure Mycobacterium tuberculosis blood stream infection: A diagnostic and disease biomarker cohort study. Lancet Microbe. 2022;3(7):e521–e532. 10.1016/S2666-5247(22)00062-3 35644157 PMC9242865

[15] Cummings MJ, Bakamutumaho B, Jain K, et al. Brief report: Detection of urine lipoarabinomannan is associated with proinflammatory innate immune activation, impaired host defense, and organ dysfunction in adults with severe HIV-associated tuberculosis in Uganda. J Acquir Immune Defic Syndr. 2023;93(1):79–85. 10.1097/QAI.0000000000003159 36701194 PMC10079575

[16] Peetluk LS, Rebeiro PF, Cordeiro-Santos M, et al. Lack of weight gain during the first 2 months of treatment and human immunodeficiency virus independently predict unsuccessful treatment outcomes in tuberculosis. J Infect Dis. 2020;221(9):1416–1424. 10.1093/infdis/jiz595 31724035 PMC7137883

[17] Khan A, Sterling TR, Reves R, Vernon A, Horsburgh CR. Lack of weight gain and relapse risk in a large tuberculosis treatment trial. Am J Respir Crit Care Med. 2006;174(3):344–348. 10.1164/rccm.200511-1834OC 16709935

[18] Wilson D, Moosa MS, Cohen T, Cudahy P, Aldous C, Maartens G. Evaluation of tuberculosis treatment response with serial C-reactive protein measurements. Open Forum Infect Dis. 2018;5(11):ofy253. 10.1093/ofid/ofy253 30474046 PMC6240901

[19] Belay M, Tulu B, Younis S, et al. Detection of mycobacterium tuberculosis complex DNA in CD34-positive peripheral blood mononuclear cells of asymptomatic tuberculosis contacts: An observational study. Lancet Microbe. 2021;2(6):e267–e275. 10.1016/S2666-5247(21)00043-4 34100007 PMC8172384

[20] Kim JW, Bowman K, Nazareth J, et al. PET-CT-guided characterisation of progressive, preclinical tuberculosis infection and its association with low-level circulating Mycobacterium tuberculosis DNA in household contacts in Leicester, UK: A prospective cohort study. Lancet Microbe. 2024;5(2):e119–e130. 10.1016/S2666-5247(23)00289-6 38244554

[21] Calderwood CJ, Wilson JP, Fielding KL, et al. Dynamics of sputum conversion during effective tuberculosis treatment: A systematic review and meta-analysis. PLoS Med. 2021;18(4):e1003566. 10.1371/journal.pmed.1003566 33901173 PMC8109831

[22] Shah M, Martinson NA, Chaisson RE, Martin DJ, Variava E, Dorman SE. Quantitative analysis of a urine-based assay for detection of lipoarabinomannan in patients with tuberculosis. J Clin Microbiol. 2010;48(8):2972–2974. 10.1128/JCM.00363-10 20534796 PMC2916614

[23] Lawn SD, Gupta-Wright A. Detection of lipoarabinomannan (LAM) in urine is indicative of disseminated TB with renal involvement in patients living with HIV and advanced immunodeficiency: Evidence and implications. Trans R Soc Trop Med Hyg. 2016;110(3):180–185. 10.1093/trstmh/trw008 26884498 PMC4755427

[24] Wood R, Racow K, Bekker LG, et al. Lipoarabinomannan in urine during tuberculosis treatment: Association with host and pathogen factors and mycobacteriuria. BMC Infect Dis. 2012;12:47. 10.1186/1471-2334-12-47 22369353 PMC3349560

[25] Drain PK, Gounder L, Grobler A, Sahid F, Bassett IV, Moosa MY. Urine lipoarabinomannan to monitor antituberculosis therapy response and predict mortality in an HIV-endemic region: A prospective cohort study. BMJ Open. 2015;5(4):e006833. 10.1136/bmjopen-2014-006833 PMC4401837 25877271

[26] Kroidl I, Clowes P, Reither K, et al. Performance of urine lipoarabinomannan assays for paediatric tuberculosis in Tanzania. Eur Respir J. 2015;46(3):761–770. 10.1183/09031936.00003315 26113682

[27] Gao Q, Zeng Q, Wang Z, et al. Circulating cell-free DNA for cancer early detection. Innovation. 2022;3(4):100259. 10.1016/j.xinn.2022.100259 35647572 PMC9133648

[28] Diehl F, Schmidt K, Choti MA, et al. Circulating mutant DNA to assess tumor dynamics. Nat Med. 2008;14(9):985–990. 10.1038/nm.1789 18670422 PMC2820391

[29] Botezatu I, Serdyuk O, Potapova G, et al. Genetic analysis of DNA excreted in urine: A new approach for detecting specific genomic DNA sequences from cells dying in an organism. Clin Chem. 2000;46(8 Pt 1):1078–1084. 10.1093/clinchem/46.8.1078 10926886

